# Rapid jail-based implementation of overdose education and naloxone distribution in response to the COVID-19 pandemic

**DOI:** 10.1186/s40352-024-00283-8

**Published:** 2024-06-27

**Authors:** Carrie B. Oser, Margaret McGladrey, Marisa Booty, Hilary Surratt, Hannah K. Knudsen, Patricia R. Freeman, Danelle Stevens-Watkins, Monica F. Roberts, Michele Staton, April Young, Emma Draper, Sharon L. Walsh

**Affiliations:** 1 Department of Sociology, Center for Health Equity Transformation, Center on Drug & Alcohol Research, University of Kentucky, 1531 Patterson Office Tower, Lexington, KY 40506 USA; 2 Department of Health Management and Policy, Center for Innovation in Population Health, University of Kentucky, 111 Washington Avenue, Lexington, KY 40508 USA; 3 Department of Sociology, University of Kentucky, 1515 Patterson Office Tower, Lexington, KY 40506 USA; 4 Department of Behavioral Science, Center on Drug & Alcohol Research, University of Kentucky, 845 Angliana Avenue, Lexington, KY 40508 USA; 5 Department of Pharmacy Practice & Science, Center for the Advance of Pharmacy Practice, University of Kentucky, 789 S. Limestone, Lexington, KY 40508 USA; 6 Department of Educational, School, and Counseling Psychology, Center on Drug & Alcohol Research, University of Kentucky, 103 Dickey Hall, Lexington, KY 40506 USA; 7 Substance Use Priority Research Area, University of Kentucky, 845 Angliana Avenue, Lexington, KY 40508 USA; 8 Department of Behavioral Science, Center on Drug & Alcohol Research, University of Kentucky, 1100 Veterans Drive, Medical Behavioral Science Building, Lexington, KY 40536 USA; 9 Department of Epidemiology and Environmental Health, Center on Drug & Alcohol Research, University of Kentucky, 111 Washington Ave, Lexington, KY 40508 USA; 10 Department of Psychology, Williams College, 25 Stetson Ct., Williamstown, MA 01267 USA

**Keywords:** COVID-19 pandemic, Naloxone, Implementation science, Jails, Practical, robust implementation and sustainability model (PRISM)

## Abstract

**Background:**

People incarcerated in jails are highly impacted by the opioid epidemic, and overdose education and naloxone distribution (OEND) is an effective strategy to reduce opioid overdose deaths. This study examines barriers and facilitators of fast-track OEND implementation within the jails in the Wave 1 Kentucky counties of the HEALing Communities Study during the COVID-19 pandemic.

**Methods:**

Meeting minutes with jail stakeholders were qualitatively coded using the Practical, Robust Implementation and Sustainability Model (PRISM) as the coding framework. The analysis highlighted the top barriers and facilitators to fast-track OEND implementation within the PRISM framework.

**Results:**

Space and staffing shortages related to the COVID-19 pandemic, disruptions in interorganizational programming from pandemic-related service suspensions, and a lack of technological solutions (e.g., reliable Internet access) for socially distanced delivery were the top barriers to fast-track OEND implementation. In addition, there were limitations on non-jail staff access to jails during COVID-19. Top facilitators included jail leadership support, the option to prioritize high-risk groups, and the incorporation of OEND processes into existing communications and management software. While the COVID-19 pandemic strained jail infrastructure, jail and partner agency collaboration led to creative implementation strategies for the successful integration of OEND into jail operations. Urban jails were more likely than rural jails to be early adopters of OEND during the public health emergency.

**Conclusions:**

Understanding the barriers to and facilitators of OEND within jails will improve implementation efforts seeking to curb opioid overdose deaths. Jail leadership support and interorganizational efforts were key facilitators to implementation; therefore, it is recommended to increase buy-in with multiple agencies to promote success. Challenges brought on by COVID-19 have resulted in a need for innovative solutions for implementation.

**Clinical trial information:**

ClinicalTrials.gov, NCT04111939, Submitted 30 September 2019, https://clinicaltrials.gov/study/NCT04111939?titles=HEALing%20Communities%20Study&rank=1.

## Background

Drug overdoses have emerged as one of the largest and most persistent public health challenges in the United States (U.S.) over the past decade. A significant proportion of people who are incarcerated for drug-related offenses or other crimes have an opioid or other substance use disorder (SUD) (Degenhardt et al., 2019; Institution for Health Metrics and Evaluation, 2021; Krausz et al., 2021). Almost one-third of individuals incarcerated in U.S. jails have an opioid use disorder (OUD) (Fazel et al., 2017), and drug overdoses are the leading cause of death among people who were formerly incarcerated, with opioids being the most common drug class involved (Binswanger et al., 2013; Lim et al., 2012; Merrall et al., 2010; Ranapurwala et al., 2018). The highest risk for overdose occurs within the first two weeks after a person’s release from a correctional institution due to reduced tolerance (Binswanger et al., 2007, 2013; Farrell & Marsden, 2008; Krawczyk et al., 2017; Krinsky et al., 2009; Merrall et al., 2010; Winter et al., 2016), indicating a critical need for the integration of overdose prevention services into reentry processes.

The provision of overdose education and naloxone distribution (OEND) to people at discharge from jail is one proven strategy for reducing opioid overdose fatalities (Grella et al., 2021), as jails provide services to highly impacted populations. Naloxone, both in intranasal and injectable forms, is a short-acting opioid antagonist medication that can safely reverse the effects of an opioid overdose when administered promptly (Giglio et al., 2015) and has no abuse potential. In the past decade, U.S. legislative support in the U.S. for naloxone distribution and bystander use has grown such that currently all 50 states and the District of Columbia have passed naloxone access laws (Prescription Drug Abuse Policy System, 2022), which are associated with increased distribution of naloxone and reductions in overdose fatalities (Smart et al., 2021). Most recently, in 2023, the U.S. Food and Drug Administration approved Narcan nasal spray for over-the-counter status (US Food & Drug Administration, 2023).

Jail-based OEND includes providing education to ensure a person knows how to recognize and respond to an overdose event (e.g., through video-based training or individual/group sessions) and distributing free naloxone to people from correctional facilities at discharge. Jail jurisdictions in the U.S. implementing OEND programs have highlighted successes (Anthony-North et al., 2018; Grella et al., 2021; Horton et al., 2017; McDonald et al., 2017; Wenger et al., 2019). For example, data from the San Francisco County jail demonstrate that 32% of the individuals who received OEND before release used it to reverse an overdose (Wenger et al., 2019). Although available data indicate that some jurisdictions in the U.S. may provide naloxone to those incarcerated while in prison or at release (Stone & Shirley-Beavan, 2018), implementation is rarely systematic or universal (Sander et al., 2019), thus limiting the potential public health impact of jail-based OEND.

### COVID-19 and jails

In early 2020, the world experienced the onset of the COVID-19 pandemic, which presented new challenges to jails and for people with OUD. Jails faced rapid COVID-19 transmission due to the limited ability to social distance and isolate COVID-positive individuals (Akiyama et al., 2020; Kim et al., 2022; Wallace et al., 2020). Individuals incarcerated in jails often have high-risk chronic conditions that make them vulnerable to contracting COVID-19 and for experiencing poor outcomes after infection (LeMasters et al., 2022; Maruschak et al., 2015; United Nations News, 2021). Thus, following the May 2020 guidance from the World Health Organization and other United Nations agencies, many jurisdictions in the U.S. implemented policies allowing for large-scale early release of persons who were charged or convicted of non-violent crimes or were medically fragile (Nowotny et al., 2020; Waly et al., 2020). While these mitigation measures reduced the spread of COVID-19, individuals with OUD missed opportunities for assessment and linkage to treatment. Moreover, SUD treatment slots were limited in many communities, and individuals who were in active recovery may have been compelled to quarantine in risky social environments. The dual epidemics resulted in an increase in overdose deaths in Kentucky (Slavova et al., 2021) and across the U.S. (Collins et al., 2020; Faust et al., 2021; Friedman & Akre, 2021), pointing to the urgency of implementing evidence-based practices (EBP) to reach those at high risk of opioid overdose.

### PRISM framework

The Practical, Robust Implementation and Sustainability Model (PRISM) is a comprehensive framework for translating research into practice, or in this case, implementing OEND programs in jails during COVID-19. According to Feldstein and Glasgow (2008), the major PRISM domains affecting EBP implementation include: *the intervention* (from the perspective of both the organization and the client/patient), *external environment*, *implementation and sustainability infrastructure*, and *recipients* (including both the characteristics of the organization and the client/patient). Each PRISM domain is likely to affect the implementation progress of an EBP in jail environments. The *intervention*, OEND, must align with the mission of the jail, which may be challenging because jails are tasked primarily with custody and protecting public safety. However, highlighting the public health importance of OEND in reducing loss of human life, cost-savings (Townsend et al., 2020), and mitigation of potential lawsuits from deaths could heighten the relevance of OEND to the jail’s mission. When considering OEND from the jails’ vantage point, organizational readiness for the intervention, OEND usability (i.e., perceived usefulness and ability to meet the jails’ needs), the ease with which OEND can be tried (i.e., trialability), cost, and the extent to which coordination across departments is needed may impact the success of the implementation process.

Elements within the *external environment* are noted by Feldstein and Glasgow (2008) as being some of the most powerful predictors of successful implementation. The impact of COVID-19 on operations cannot be overstated within jail environments. Interorganizational efforts and relationships of quality, value, and trust between the jail and other organizations could enhance the provision of OEND. Moreover, Kentucky jailers and other local officials (i.e., fiscal court judge executives) who allocate funding to jails are elected; therefore, the level of local community support for OEND and people with OUD could influence decision-making by these elected officials. Legal concerns related to the provision of naloxone, a prescription medication at the time of the present study, is another external factor that could hinder OEND implementation.

Regarding the *recipient* PRISM domain, characteristics of the jail may affect OEND implementation. Leadership support is critical, as jailers often influence organizational culture and serve as change agents. Clear communication of support for an OEND program from top leaders to front-line staff can build the necessary buy-in for success. Another element that may influence implementation includes systems and training, defined as the jail infrastructure and operations available to run an OEND program. Staffing concerns, such as the lack of available staff and the burden on staff to run an OEND program, may exist. To maximize the reach of jail-based OEND programs, the characteristics of the people who are incarcerated also must be considered (e.g., providing OEND to those in jail-based SUD treatment programs due to elevated overdose risk). Another client/patient characteristic is whether a person has been sentenced, as this impacts jail staff’s ability to reliably anticipate discharge dates for naloxone distribution.

Finally, *infrastructure* is necessary for successful implementation and sustainability. Elements within this domain include the jail’s attention to performance data, the allocation of dedicated jail staff to OEND efforts, implementation training and support, and planning for sustainability. While infrastructure can be adapted over time, it is important to consider sustainability at the beginning of the implementation process (Feldstein & Glasgow, 2008). These elements can facilitate the long-term maintenance of an OEND program within a jail.

### The current study

The unplanned early release of thousands of people to mitigate the spread of COVID-19 in the U.S. created an urgent need to provide OEND to people incarcerated in county and regional jails. Launched in 2019 with support from the National Institutes of Health and the Substance Abuse and Mental Health Services Administration, the HEALing (Helping to End Addiction Long-term^SM^) Communities Study (HCS) was designed to reduce opioid-involved overdose deaths by increasing the availability and uptake of EBPs in healthcare, behavioral health, criminal legal systems, and other community settings serving high-risk populations (Chandler et al., 2020; El-Bassel et al., 2020). Guided by the PRISM implementation science framework described in detail by Knudsen and colleagues (2020), HCS tests the Communities That HEAL (CTH) intervention comprising three components: data-driven community engagement processes to create an implementable and sustainable action plan for overdose reduction that addresses local needs; implementation of community-selected EBP strategies to increase OEND, improve delivery of medication for opioid use disorder treatment, and advance prescription opioid safety; and creation and deployment of communication campaigns to reduce stigma and promote EBPs in the community (Sprague Martinez et al., 2020; Walsh et al., 2020; Winhusen et al., 2020).

When the COVID-19 pandemic hit the U.S. in the initial months of the CTH intervention, the HCS was poised to explore the implementation of OEND in criminal legal settings during the public health emergency using an expedited protocol dubbed “fast-track.” There are no known studies examining OEND implementation in jails during the pandemic (Nowotny et al., 2020). Thus, the purpose of this qualitative study is to describe the process of fast-track implementation of OEND for all individuals released from HCS partner jails during the COVID-19 pandemic and to identify facilitators and barriers (both related and unrelated to COVID-19) through the lens of PRISM (Feldstein & Glasgow, 2008).

## Methods

### Study design

HCS is a multi-site, parallel-group, cluster randomized wait-list controlled trial conducted in 67 communities (34 in active intervention and 33 in wait-list control) in Kentucky, Massachusetts, New York, and Ohio that are highly burdened with opioid overdose and diverse in terms of rural-urban status and race/ethnicity (El-Bassel et al., 2020). Communities were randomly assigned to either the CTH intervention (Wave 1 communities) or the wait-list comparison group (Wave 2) per the HCS protocol’s covariate-constrained randomization method (Walsh et al., 2020). The CTH intervention was implemented in Wave 1 communities from January 2020 to June 2022. This study is registered on Clinical Trials.gov (https://clinicaltrials.gov/ct2/show/NCT04111939) and was approved (Pro000308088) by Advarra Inc., the HEALing Communities Study single-Institutional Review Board.

### HCS fast-track protocol

When the early response to the pandemic amplified risks related to opioid overdoses, the HCS developed a “fast-track” protocol allowing for expedited implementation of OEND to high-risk populations through direct financial support for OEND and technical assistance for agencies implementing OEND. HCS community coalitions were presented with the option to approve the expedited launch of OEND efforts in jails (Young et al., 2022). Kentucky was the only one of the four participating HCS research sites to directly partner with jails to provide and track no-cost naloxone for distribution at release, so this study focuses on the implementation of the OEND fast-track protocol in HCS-Kentucky Wave 1 regional/county jails.

After coalition approval, an HCS-KY Implementation Facilitator reached out to jail leadership via email or phone to set up an introduction meeting; in some cases, HCS coalition members or key government stakeholders provided introductions. The Implementation Facilitator worked with agencies through a series of structured meetings to develop implementation plans tailored to a jail’s specific needs and leverage existing infrastructure while planning for sustainability from the start.

Kentucky’s naloxone access law, Ky. Rev. Stat. § 217.186, was used to guide the development of standard operating procedures for dispensing naloxone to partner agencies, including jails. Agencies wishing to participate entered into a standing order agreement (SOA) with an HCS-affiliated physician. The SOA authorized the dispensing of NARCAN^®^ Nasal Spray 4 mg at no cost to the partner agency, which then could distribute HCS-provided naloxone to any person who received training in specific elements of overdose prevention and response. The SOA further required documentation of dispensing and distribution by both HCS and the partner agency (see Knudsen et al., 2023 for additional details).

Although the elements of the OEND process were required to align with the terms of the SOA, individualized implementation designs were necessary to account for varying settings and workflows across partner agencies, including jails. The required overdose education (OE) could be completed through live one-on-one or group interactions, an HCS-developed training video, or an online interactive module. Similarly, documentation of distribution and demographics could occur directly via entry into HCS’s Research Electronic Data Capture (REDCap) (Harris et al., 2009) database using an HCS-supplied tablet computer or could be provided to HCS on paper forms, which were entered into the REDCap database manually by HCS staff.

### Setting

The eight Wave 1 Kentucky communities represented diverse implementation settings, with four counties classified as rural and four as urban by the U.S. Department of Agriculture’s Rural-Urban Continuum Codes (RUCC) (United States Department of Agriculture, 2020; Walsh et al., 2020). The average daily census of the regional and county jails in these eight Kentucky counties also varied widely, from 132 to 898 (Vera, 2022), but specific census and demographics of the jail population are not reported to protect the jails’ identities. As the COVID-19 pandemic began, the Kentucky Justice and Public Safety Cabinet followed the Centers for Disease Control and Prevention (2022) COVID-19 guidance for correctional and detention facilities to prioritize early release for those vulnerable to COVID-19 because of a health condition and nearing the end of their sentence while excluding those convicted of sexual and violent crimes.

### Data sources

From March 2020 to December 2020, meeting minutes were recorded for every Zoom meeting that occurred between the HCS research team (composed of faculty, Implementation Facilitator, and other research staff) and jail partners to plan implementation of the fast-track OEND protocol for individuals being released from jails. Meeting minutes followed a structured outline for consistency across the eight Wave 1 jails and were recorded by a trained research team member who was in attendance. For example, the Implementation Facilitator used the meeting guide which included introductions, a brief overview of the HCS aligning with a PowerPoint presentation, and a discussion of the possibility of partnering on OEND. The discussion covered current overdose education efforts and possible strategies for distributing naloxone to the general jail population in light of COVID-19 restrictions. When possible, descriptions of the conversations included attribution to specific speakers with occasional direct quotes included, and minutes were recorded in a bulleted format. The meeting minute documents were labeled by meeting date and jail location by a research assistant and uploaded to NVivo 12 for coding and analysis. To identify factors influencing the initial implementation of the OEND program in the context of the pandemic, only minutes for meetings proceeding each jail’s first date of naloxone distribution were analyzed.

### Analytic plan

We utilized PRISM domains and elements to develop the coding structure, engaging in directed/deductive coding (Hsieh & Shannon, 2005). A five-person team comprising three faculty and two research assistants trained in qualitative research methods conducted directed content analysis. The first step of the process entailed the research assistants open coding meeting minutes to assess whether PRISM elements applied to this dataset and whether categories appeared that were not captured by the PRISM framework. COVID-19 is not specifically defined by PRISM, but the coding team added a COVID-19 code to the codebook for analyzing challenges and innovations that stemmed uniquely from pandemic restrictions.

After the coding team finalized the codebook, they undertook consensus coding to ensure consistency in interpretation, whereby two of the faculty coders randomly selected 20% of the meeting minutes and compared their code applications to those of the research assistants. When inconsistencies were identified, the team discussed their reasoning and came to a consensus on a consistent interpretation, or if there was a tie, the majority ruled. A research assistant re-coded any discrepancies and then applied the finalized codebook to the remaining meeting minute documents using NVivo 12. Finally, a research assistant applied “barrier” and “facilitator” codes to all meeting materials classified with PRISM and COVID-19 codes to assign valences denoting whether the PRISM and/or COVID-19 element presented obstacles or supported OEND implementation.

## Results

Jails in five of the eight HCS Wave 1 communities implemented OEND, meaning they distributed naloxone units to people who were released from incarceration within the fast-track timeline (March-December 2020) (see Table 1). No jails provided OEND to all persons at discharge before involvement in HCS. The structure of each jail’s OEND program varied to address the workflow needs of the jail. For example, infrastructure for staff-led OEND existed or was created in some jails, while other jails utilized recorded training videos or collaborated with the local health department or a recovery community organization (RCO) for implementation. Table 1 shows the details of each jail’s OEND program, OEND implementation timelines, the number of meetings that occurred before the first naloxone distribution, and the average number of jail staff in attendance.

**Table 1:** Jail characteristics and overdose education & naloxone distribution (OEND) implementation timelines within the fast-track protocol timeframe (march to December 2020)

Jail & RUCC classification	OEND structure	Standing order agreement (SOA) Date	Month/year of first naloxone distribution	# Of months between SOA & date of first naloxone distribution	# Of Meetings before first naloxone distribution	Average number & range of jail staff attending meetings
*Early Adopters*						
Jail #1 Urban	Naloxone dispensed to jail; jail staff provide in-person training during incarceration and distribution at release	4/3/2020	4/2020	0	2	Average = 1.5 Range = 1–2
Jail #2 Urban	Naloxone dispensed to Recovery Community Organization that goes on-site at jail to provide in-person training and distribution at release	4/8/2020	5/2020	1	2	Average = 1 Range = 1
Jail #3 Urban	Naloxone dispensed to jail; jail staff provide in-person training during incarceration and distribution at release	5/14/2020	6/2020	1	2	Average = 1.5 Range = 0–3
*Late-Adopters*						
Jail #4 Urban	Naloxone dispensed to jail; education provided during incarceration via HCS video on tablets available to people incarcerated within the jail; distribution at release	4/16/2020	11/2020	7	3	Average = 3 Range = 2–5
Jail #5 Rural	Naloxone dispensed to local health department that goes on-site via a mobile unit (staffed by both HCS and health department) at the jail to provide in-person training and distribution at release	6/19/2020	10/2020	4	1	Average = 2 Range = 2
*Non-Adopters*						
Jail #6 Rural	Naloxone dispensed to jail; education provided during incarceration via HCS video on tablets accessible to people who are incarcerated; distribution at release	4/2/2020	4/21* (OEND program began after the fast-track protocol timeframe)	12*	6* (5 meetings without naloxone distribution)	Average = 2.4 Range = 1–4
Jail #7 Rural	Naloxone dispensed to jail; jail staff provide in-person training during incarceration and distribution at release	4/16/2020	6/22* (OEND program began after the fast-track protocol timeframe)	26*	8* (5 meetings without naloxone distribution)	Average = 1.8 Range = 0–3
Jail #8 Rural	OEND not implemented	n/a	n/a	n/a	n/a (5 meetings without naloxone distribution)	Average = 0.4 Range = 0–1

*NOTE OEND program began after the fast-track protocol timeframe, which ended on 12/31/20

As illustrated in Table 1, all “early-adopter” jails (*n* = 3, those for which it took 3 months or less for naloxone distribution to begin after the SOA was signed) were in counties classified by the RUCC as urban. Of the two “late-adopter” jails (those for which it took more than 3 months for naloxone distribution to begin after the SOA was signed), one was in a rural county, while the other was classified as urban. All “non-adopter” jails were in rural counties (*n* = 3, those with which HCS met to facilitate fast-track OEND implementation that either did not complete the SOA or did not distribute naloxone after completing the SOA within the “fast-track” timeframe ending on December 31, 2020). In general, HCS facilitated more pre-implementation planning meetings with rural than with urban jails during the rural jails’ longer implementation timelines.

It should be noted that the workflow for rural jail #5, a late adopter, was revised after the HCS fast-track timeframe to place additional staff into the jail. This workflow was replicated for use in rural jails #6 and #7 which did not implement OEND during the fast-track timeline but did implement during the longer HCS intervention period. Specifically, a certified peer-support specialist employed by a partnering RCO or a social work navigator employed by a partnering healthcare agency was deployed full-time to the jail to provide additional services, including OEND. These new HCS-funded staff positions allowed for OEND implementation, though the option and need to identify, hire, and train new staff for deployment exceeded the fast-track timeline. Rural jail #8 did not implement an OEND program.

Most jails did not need an on-site naloxone supply from HCS for jail staff to respond to overdoses within the facility, as they already had a small supply for on-site administration through either the state government or their local health department. Several jails responded to overdoses within their facilities during the fast-track period of March-December 2020, reiterating the need for both on-site supplies and naloxone distribution to trained people at release. After the fast-track timeline, rural jails #5 and #7 requested an on-site HCS naloxone supply to ensure they were adequately equipped.

### Overview of most prevalent PRISM domains and elements

Table 2 provides an overview of the operationalization of PRISM elements within the qualitative codebook. It should be noted that 29 total PRISM element codes were included in the codebook and applied in the analyses; however, only those discussed in this paper are included in Table 2. The term “prevalence” is used for a more robust presentation of the elements that emerged as key themes from the qualitative data.

**Table 2:** Operationalization of PRISM element definitions for codebook

PRISM Domain	Element	Codebook Definition
Intervention – perspective of the organization (jail)	Readiness for change	The extent to which organizational members are psychologically and behaviorally prepared to implement organizational change” (Weiner et al., 2008). Code when willingness (or lack thereof) to implement OEND strategies is apparent among agency members.
	Trialability	The ability to try the program; code when discussion of implementation plan relates to integrating the intervention into organizational functioning or staff duties.
External environment	COVID-19 pandemic	References to any discussion of impact of the COVID-19 pandemic on OEND implementation.
	Interorganizational efforts	Related to discussion of interorganizational networks’ relationship quality, value, and trust.
Recipient characteristics – jail and people incarcerated	Leadership support	Refers to expressions of leadership support (e.g., jailers) of implementation. Endorsement of ideas, rather than actionable tasks.
	Staffing concerns	Refers to mentions of constraints on staff time and ability to facilitate OEND.
	Systems and training	Refers to discussions of OEND infrastructure in place pre-HCS implementation.

*Notes* This table includes only codebook definitions for the PRISM elements referenced herein that emerged as key themes; there were 29 total PRISM element codes defined for this study’s codebook and applied in the analysis

Table 3 outlines the top five most frequently used PRISM element codes applied as barriers and as facilitators, which aligned with only three PRISM domains: *organizational perspectives on the intervention, recipient characteristics*, and *external environment*. Key findings for each of these three PRISM domains are included in italicized text in Table 3 to highlight factors critical to OEND implementation in jails. None of the element codes within two domains (i.e., perspectives of *recipients* on the intervention and *implementation and sustainability infrastructure*) surfaced among the top-five overall barriers and facilitators to OEND uptake. This pattern indicates that some PRISM domains were not as relevant as others to this implementation context. Each of these PRISM domains and elements is described below with detailed examples and illustrative quotes based on if they were a barrier only, a facilitator only, or both a barrier and a facilitator to OEND implementation progress.

**Table 3:** Summary of prevalent PRISM domains and elements with barrier and facilitator valences with key findings

Barriers: Most frequently used PRISM element codes and key findings	Facilitators: Most frequently used PRISM element codes and key findings
**1. COVID-19 pandemic (19.4%)**	**1. Trialability (14.6%)**
• Disrupted or suspended programming and screening practices • Limitations on non-jail staff access to facilities	• Option to prioritize high-risk groups • Easy integration into jail management software
2. **Trialability (12.0%)**	2. **Leadership support (12.5%)**
• Space and staffing shortages reduce the ability to try out OEND	• OEND buy-in driven by the severity of the opioid overdose crisis among people incarcerated and the experiences of peer jailers with successful adoption
3. **Systems and training (10.2%) **	3. **Interorganizational efforts (11.4%) **
• Limited infrastructure for technological solutions (e.g., reliable Internet access)	• Partnerships with local community agencies allowed for OEND • Contracts with communications companies facilitated OEND
4. **Interorganizational efforts (9.9%) **	4. **Readiness for change (9.0%) **
• Disruptions to existing partnerships with local community agencies due to the pandemic	• Technological capacity allowed for socially distanced overdose education • Jailers’ commitment to addressing stakeholder misperceptions
5. **Staffing concerns (8.7%) **	5. **Staffing concerns (8.7%) **
• Staff turnover/illness • Budget cuts due to lower censuses with pandemic mass releases • High volumes of releases strained staff capacity	• Technological infrastructure supports OEND • Staff with behavioral health/re-entry expertise support OEND efforts

*Notes* % = frequency of PRISM element code usage across total # of PRISM element codes (matrix-coded by barrier and facilitator). Key findings are in italicized text

### PRISM elements with only barrier valences

#### COVID-19 pandemic as an OEND implementation barrier

The most prevalent barrier identified to jails’ OEND implementation efforts involved the PRISM external environment domain. The primary barrier elicited in this domain was the pandemic itself, which served as the catalyst for the expedited OEND protocol in the jail setting but simultaneously presented numerous logistical barriers to implementation. By the time of the study team’s initial meetings with Wave 1 jails, all had begun initial releases of people who were incarcerated in their facilities per orders from the state and were experiencing several COVID-related impacts on their typical operations. Several jail leaders mentioned that overarching COVID-19 safety guidance issued by the Justice and Public Safety Cabinet had affected their routine processes for medical and substance use screening among individuals incarcerated. Programming locations were no longer available due to physical distancing requirements, essentially pausing all jail-based programming. Moreover, many jails shared that they were no longer able to admit any non-jail staff, such as volunteers or research staff, due to the pandemic. This constellation of factors represented a limitation on autonomy to implement OEND for jails and constrained their ability to freely integrate OEND into existing programming and workflows.

#### Staffing concerns as an OEND implementation barrier

Staffing concerns at the detention centers emerged as a major barrier to expedited OEND implementation. Importantly, jail leaders noted that staffing levels are typically challenging due to turnover and low pay for correctional officers; however, this was exacerbated during COVID-19. Most jail leaders mentioned staffing as a concern for launching OEND, indicating that their staffing levels had fallen significantly due to COVID-19 exposure and/or illness among employees, or they had experienced systematic budget cuts due to lower census levels in the facilities. For example, in early April 2020, one rural jailer reported that his facility had gone from more than 400 to 230 people incarcerated in the prior three weeks. A jail leader explained:

Due to the reduction in number of inmates, staffing hours have been cut as well. Every current staff member has lost hours at work, and the majority are part-time. The jail is covered financially by how many inmates they have in current custody; therefore, as inmates decrease, their financial support for the jail decreases and so does working time allotted to personnel.

These pandemic-related staffing challenges created notable limitations on jail bandwidth to provide OEND. The sheer volume and rapid, unpredictable nature of releases occurring (e.g., uncertainty about how many people might be released each week due to medical conditions) represented unsupportable strains on existing staff. Compounded by prohibitions to jail entry by other personnel noted earlier, support for successful OEND implementation required strategies with minimal touchpoints by jail personnel to reduce staff burden.

### PRISM elements with only facilitator valences

#### Leadership support as an OEND implementation facilitator

As a facilitator, the second-most frequently coded element overall was leadership support for OEND implementation. A clear indicator of leadership support was jailer attendance at HCS planning meetings during which they voiced their interest in and commitment to OEND implementation with their staff in attendance. For some jail staff, the life-and-death stakes of the opioid overdose crisis were strong motivations for OEND implementation; one rural jailer said, *“In a seven-day period, we had five people who were released who passed away. We thought that was eerie, and we thought we had a problem at the jail. We got in touch with the coroner and learned they had overdosed.”* Another rural jailer mentioned an incident in 2019 when an overdose occurred in the facility and his staff could not find naloxone, noting that he never wants this to happen again. One urban jailer in an early-adopting jail extended an invitation to a meeting with another rural jailer to share tips and lessons learned in his facility’s successful OEND implementation. Jailer buy-in to OEND was driven by the severity of the opioid overdose crisis among the people in or leaving their custody and by peer jailers’ successes with implementation.

#### Readiness for change as an OEND implementation facilitator

Defined as apparent willingness among agency staff to implement OEND, readiness for change was the fourth-most prevalent element coded as a facilitator. Readiness for change was evident in discussions of how tablets and communication system contracts could be used to facilitate OE in a way that did not require staff and groups of people who are incarcerated to be in close proximity, reducing COVID-19 risk. In addition, readiness for change was evident when jail leadership described their buy-in to OEND resulting from direct experience of individuals dying from overdoses immediately after release and/or while in their facilities. An example of leadership support also coded as readiness for change was an urban jailer describing a weekly meeting with law enforcement and emergency management personnel during which concerns were raised about OEND in the jail related to liability. The jailer’s readiness for change was evident in his willingness to correct misperceptions of who is eligible by Kentucky law to carry naloxone, thereby encouraging increased readiness among relevant local stakeholder agencies who might encounter individuals carrying and using naloxone in the community after re-entry. Readiness for change was enhanced by jails’ technological capacity to facilitate socially distanced OE and jail leadership’s confidence in addressing myths among their counterparts about naloxone use, toward their goal of reducing overdoses in their facilities and after release.

### PRISM elements with both barrier and facilitator valences

#### Trialability as an OEND implementation facilitator and barrier

Trialability, defined as the jail’s ability to integrate OEND into existing organizational functioning to try out the EBP, was frequently coded as both a barrier and facilitator to implementation. As noted above, infrastructure constraints and space limitations that existed pre-pandemic became operational obstacles during COVID-19. For example, a small office in a rural jail that had previously been used to meet with people who were incarcerated could not be used to provide OE training because of social distancing requirements. Additionally, trialability was intertwined with staffing and bandwidth concerns, illustrated by jail leadership’s frequent mentions that the release process involves too many steps to integrate another discrete task (i.e., OEND). Jail space and staffing constraints presented challenges to trying out OEND, especially in the context of social distancing.

One of the most salient aspects of trialability as a facilitator was the ability to try the intervention with specific subsets of people who were incarcerated, for example, only individuals who had been sentenced or individuals already receiving SUD-related services. Multiple jails noted this targeted approach, rather than universal OEND, would result in a more manageable initial workload, offer more predictable notice of release dates, and better leverage staff in health-related or re-entry-focused roles rather than adding burden to correctional officers. Another example of trialability as a facilitator was the suggestion by one rural jail for a “soft open” for OEND by using ancillary spaces in the lobby or outside, providing an opportunity to try out the process without disrupting core jail operations. A final example of trialability as a facilitator was when urban jail staff discussed their ability to use the existing “hold” function on their information management system called JailTracker™ to flag individuals who had completed OE and should receive naloxone at release. OEND implementation was supported by trial periods with specific groups of people who were incarcerated and in specific areas of jail facilities as well as by OEND integration into management software.

#### Interorganizational efforts as an OEND implementation facilitator and barrier

In the external environment domain of PRISM, interorganizational efforts were defined for this study as references to high-quality operational relationships between jails and other organizations serving the community. Interorganizational efforts were frequently coded as both a barrier and facilitator to jail implementation of OEND. As a barrier, interorganizational efforts manifested in various ways. Jail leadership mentioned existing partnerships with local community agencies such as health departments, recovery community organizations (RCOs), and SUD treatment organizations to offer various services to people who are incarcerated; however, without exception, these services had paused due to COVID-19. As a result, despite the presence of productive partnerships and general openness to collaborations, the ability to leverage existing workflows for integration of OEND was severely limited. For example, staff in one urban jail noted that all services provided by the local health department in their facility (which included OEND to a subset of people) were suspended during the pandemic. Suspension of services provided by outside organizations during a time of exceedingly high need left many individuals abruptly returning to the community vulnerable to overdose.

However, COVID-19 did not preclude the utility of interorganizational efforts as a facilitator to OEND implementation when partnerships could be established without the other agency staff entering the jail’s secured units. For example, one urban jail mentioned their amenability to partnering with a local RCO to offer OEND while pandemic-related staffing challenges limited jail bandwidth to directly provide it. In this model, the jail would notify the RCO of releases and RCO staff would go to the jail to meet the person at their release for OEND. Another example was of an urban jailer who worked with his county attorney to determine whether Ky. Rev. Stat. § 441.127 would allow for a one-day reduction in sentences for people who completed OE to incentivize participation. This interorganizational effort did not come to fruition during the “fast-track” timeframe but was illustrative of innovative collaboration.

Several jails had contracts in place with communications companies providing tablets and educational content to individuals incarcerated in their facilities. These external relationships allowed HCS to partner with the communication vendor to load the interactive OE course created by HCS into the tablet programming and with the jailer’s decision to make its completion mandatory, place OE in the “favorites” section of the tablets, and/or display a message about OE each time an individual logs onto the tablet in four jails. However, as noted in the systems and training discussion below, the utility of this facilitator was sometimes reduced by the lack of information technology (IT) support and challenges with Internet access in the facilities. These findings indicate jails’ general reliance on interorganizational efforts, which were undermined by pandemic restrictions on non-staff entry into facilities and could be affected by Internet access.

#### Systems and training as an OEND implementation facilitator and barrier

The third-most prevalent barrier and the fifth-most prevalent facilitator, the systems and training element represented the infrastructure and operations in place before the HCS partnership that could support successful pathways for OEND implementation. Most frequently, jail leadership commented on barriers related to limited infrastructure for technological solutions that would support opportunities for OEND under social distancing guidelines. We observed wide variability in jails’ use of and access to technology; some lacked Wi-Fi, others lacked communications and tablet solutions, and some jail staff expressed limited experience with customizable training platforms. Access to IT support or resources was reported as a challenge, and finally, some had trouble with integration in their current JailTracker™ system. Some jail-specific technology limitations could be overcome with HCS-supported options. However, variability in jails’ comfort and experience with technology solutions was a rate-limiting step for OEND implementation in some cases.

As a facilitator, systems and training was often double-coded with trialability facilitators, such as the use of tablet technology to facilitate OE or the ability to place holds on client accounts to ensure naloxone was distributed at release to people who had completed OE while incarcerated. Other unique examples of systems and training as a facilitator included the presence of a contracted pre-release caseworker who assisted people being released with re-entry tasks and practices to systematically provide OE to each dorm every month. Although caseworker access was often disrupted by the pandemic, these existing supports for re-entry processes helped jails envision how the HCS OEND program could be integrated into their standard procedures. OEND implementation benefits from careful integration into technologies, processes, and staffing that are core to jails’ standard operations.

## Discussion

Multiple scoping reviews of OEND programs for justice-involved people have called for research to address barriers and facilitators to widespread implementation within correctional settings, including U.S. jails (Grella et al., 2021; Horton et al., 2017). This is the first known study to examine the rapid implementation of OEND for people at release from jails during the global COVID-19 pandemic. Numerous strategies were implemented in early 2020 to mitigate the spread of COVID-19 in jails (e.g., early release, limited movement/cell block isolation, suspended visitation, and pauses in services and programming); however, these operational changes also created an urgent need to provide OEND to people nearing release as the U.S. opioid overdose rate was increasing (Centers for Disease Control and Prevention, 2020; Hedegaard et al., 2021).

### Understanding barriers and facilitators affecting Jail OEND program implementation through the PRISM framework

The findings from this study point to the utility of implementation science frameworks such as PRISM (Feldstein & Glasgow, 2008) for understanding barriers and facilitators to the implementation of EBPs such as OEND in jail contexts. The PRISM framework includes both external and internal factors as well as the interplay between how the external environment may affect internal factors, such as organizational functioning, which then may affect the likelihood of innovation implementation.

Certainly, the external factor of COVID-19 was a substantial barrier to starting OEND programming in jails. Much like other settings in the OUD continuum of care, such as community-based SUD and medication for OUD treatment programs, the daily operations of these eight jails were upended by the pandemic. Similarly, community-based providers of SUD treatment faced operational challenges in the early months of the pandemic in providing services as evidenced by decreased treatment admissions (Mark et al., 2021) and patients’ reports of services being discontinued (Huhn et al., 2022).

There were notable examples of how the external factor of COVID-19 intersected with, and in some cases, worsened, long-standing organizational challenges that have been observed within jail settings. The physical distancing requirements associated with COVID-19 were particularly challenging for OEND implementation, as some jail staff felt the magnitude of this barrier made them unwilling to provide OEND on even a trial basis. Historically, jails have been overcrowded (Kim et al., 2022; Novisky et al., 2021), and while large-scale early releases helped to ameliorate overcrowding to some degree, there still were limited physical spaces in which to offer OEND. Even if sufficient staffing had been available, physical distancing requirements still would present likely challenges to implementation in several of the jail settings, particularly the jails located in rural counties with smaller facilities.

The policy response of increased early releases during the early phase of the COVID-19 pandemic had an unintended consequence in that it decreased funding to jails, which reduced available staffing. U.S. jails are operated and funded locally, which historically has resulted in heterogeneity in resources and available services (Turney & Conner, 2019). Due to prison overcrowding in Kentucky, most jails contract with the Kentucky Department of Corrections to house people under state-level custody; however, this source of revenue decreased during the pandemic due to early releases. Jails also have been environments with substantial staff turnover; for example, a national U.S. survey of jail staff found that 38% reported intentions of quitting (Leip & Stinchcomb, 2013). The financial disruption of the pandemic on funding along with staff absences due to illness and COVID-19 presented significant challenges to implementing a new program. Even without the COVID-19 pandemic, jail staff turnover would likely be a persistent issue for successful OEND implementation.

Given staffing concerns, we attempted to develop technological solutions that could reduce burdens on jail staff, but a lack of technological support proved a significant barrier in several jails. The barriers to technology reflect larger patterns in which correctional settings often have policies to limit Internet access within jails (Drabinski & Rabina, 2015; Novisky et al., 2021). There have been examples of jails that quickly pivoted to expand IT to support telehealth when they were able to reallocate grant funding (Donelan et al., 2021), but several jails in our study lacked the financial resources to scale up telehealth and expand Internet access. This technological divide remains a barrier to OEND implementation even after the harmful impacts of COVID-19 have lessened.

Although some jails were unable to overcome these barriers, more than half of the jails did implement OEND within the fast-track timeframe, and all but one jail ultimately implemented OEND during the HCS Wave 1 intervention period. The most notable facilitator of implementation was leadership support within the jail, and this factor likely remains relevant post-pandemic. This finding aligns with the broader implementation science literature that has demonstrated how leadership support is a critical ingredient in achieving successful implementation (Aarons et al., 2014; Fagan et al., 2019), particularly when leaders become champions of the implementation effort (Aarons et al., 2016; Williams et al., 2020). Leadership support was interwoven with perceptions that OEND would have a relative advantage over the status quo in that it could prevent overdose deaths within the jail, including potential legal action, as well as post-release. Perceptions of relative advantage have long been viewed as important to the innovation adoption and implementation process (Rogers, 2003).

It is noteworthy that most of the leadership support codes were applied to rural jails. From this finding, it might be inferred that for rural jails in a state where jailers are elected to their positions, leadership support is critical to OEND implementation because of the jailers’ positions of singular influence in their small, less-populated jurisdictions. However, only one of four rural jails implemented OEND within the fast-track timeframe. This highlights that while rural jailers’ support is necessary for OEND implementation, it is not sufficient to overcome other barriers to OEND in rural jails.

### Geographic location and adopter categorization

Only five of the eight jails implemented an OEND program during the HCS fast-track timeframe of March to December 2020, despite the HCS provision of an Implementation Facilitator to work with the jails on tailoring the implementation process as well as financial resources (e.g., no-cost naloxone, staffing). Using Rogers’ (2003) terminology, the three “early adopter” jails were all located in urban counties, while the three jails that did not implement OEND during the fast-track timeframe were located in rural counties. This finding highlights the significance of greater access to resources in more densely populated areas, including a larger pool of correctional officers to address staff turnover or cover shifts missed due to COVID-19 exposure or illness. Jails located in urban areas have more opportunities to collaborate with other agencies on public health initiatives such as OEND (e.g., RCOs, health departments). Additional efforts are needed in rural areas to address inequities in the provision of OEND in jails. For example, SAMHSA State Opioid Response grants or opioid abatement resources could be used to support additional staff (e.g., certified peer support specialists) to increase rural jails’ capacity to offer in-person OEND to people at discharge as well as provide OEND to visiting family and friends. This interorganizational effort facilitated three of the four rural jails’ ability to implement an OEND program with limited institutional investment and addressed administrative concerns about staff bandwidth to operate the OEND program, which is salient beyond the COVID-19 pandemic. This could serve as a model for other rural jails to provide OEND, even outside of public health emergencies.

### Limitations

Generalizability is limited, as this paper focuses on the analysis of meeting minutes with jails within the eight Wave 1 Kentucky communities in the U.S. participating in the HCS. HCS Implementation Facilitators guided the implementation process, and HCS financial resources, including staffing, were available to support the implementation of an OEND program, which may not translate to other communities interested in the rapid implementation of OEND programs in jail. In addition, this paper only focuses on the timeframe up to when the first naloxone distribution occurred, limiting the ability to describe ongoing implementation and sustainability. HCS was guided by the PRISM/RE-AIM framework (Knudsen et al., 2020), and additional research is exploring the reach of the intervention (Knudsen et al., 2023) and sustainability. In addition, the intervention for recipients (i.e., people incarcerated) was not a prominent PRISM domain, but this likely reflects the study design and the nature of data collection. Our implementation strategies only included jail staff, so it is unknown the extent to which OEND could have been more effectively implemented if input from people incarcerated had been a part of the process. However, it is also important to note that several studies have documented that many justice-involved individuals are interested in OEND and perceive it as an acceptable intervention (Barocas et al., 2015; Curtis et al., 2018; Davidson et al., 2019). Future research should consider how the delivery of OEND might be adapted to better meet the needs of incarcerated individuals by including their input during the planning process. However, this study makes a significant contribution, as there is no known literature examining varied OEND implementation programs in U.S. jails, especially during a global pandemic.

## Conclusions

Overdose deaths increased in the U.S. due to the COVID-19 pandemic, and the criminal legal system is noted as one missed touchpoint where there could be an opportunity for intervention (Tanz et al., 2022). As part of the HCS, the Kentucky team targeted this touchpoint to increase access to naloxone for people at high risk of opioid overdose being released from jails early in the COVID-19 pandemic. While OEND is an EBP, our findings indicate there are several challenges to successful OEND implementation in jails. Leadership support, such as a champion within the jail, is key for successful OEND implementation but is not sufficient for overcoming barriers in rural jails. To prevent increased overdose deaths during future public health emergencies, this study suggests that leaders within the criminal legal system (such as jailers) and policy-makers consider structural changes. To address the external environment, jails and their funders could collaborate with local health departments to provide OEND as part of the standard discharge process, with a county-level allocation of resources to this life-saving over-the-counter medication. A jail and health department partnership to implement a mail-based OEND program or co-manage a naloxone vending machine within the jail was not explored as part of this study but could be a feasible implementation strategy. This is particularly important in rural areas where existing services may be limited. In addition, scaling up technological capacity, especially within rural jails, would ease implementation efforts, allow jails to try an OEND program with limited jail staff support, and expand access to a variety of other programs and services for people who are incarcerated. In addition to saving lives, OEND programs in jails provide cost-offsets within their communities via opportunities for individuals to engage in OUD treatment and contribute to the local economy (Townsend et al., 2020). OEND within jails is essential to address opioid-related mortality in the U.S.

## Data Availability

The qualitative data analyzed during the current study are not publicly available because they are minutes of meetings from agencies partnering with the HEALing Communities Study research team. The corresponding author may share de-identified data upon reasonable request.

## Abbreviations

CTH: Communities That HEAL
EBP: Evidence-based practice
HCS: HEALing Communities Study
IT: Information technology
MOUD: Medication for opioid use disorder
OE: Overdose education
OEND: Overdose education and naloxone distribution
OUD: Opioid use disorder
PRISM: Practical, Robust Implementation and Sustainability Model
RA: Research assistant
RCO: Recovery community organization
REDCap: Research Electronic Data Capture
RUCC: Rural-Urban Continuum Codes
SOA: Standing order agreement
SUD: Substance use disorder
U.S.: United States

## References

[CR1] Aarons GA, Ehrhart MG, Farahnak LR, Sklar M (2014). Aligning leadership across systems and organizations to develop a strategic climate for evidence-based practice implementation. Annual Review of Public Health.

[CR2] Aarons GA, Green AE, Trott E, Willging CE, Torres EM, Ehrhart MG, Roesch SC (2016). The roles of system and organizational leadership in system-wide evidence-based intervention sustainment: A mixed-method study. Administration and Policy in Mental Health and Mental Health Services Research.

[CR3] Akiyama MJ, Spaulding AC, Rich JD (2020). Flattening the curve for incarcerated populations—Covid-19 in jails and prisons. New England Journal of Medicine.

[CR4] Anthony-North, V., Pope, L. G., Pottinger, S., & Sederbaum, I. (2018). *Corrections-based responses to the opioid epidemic: Lessons from New York State’s overdose education and naloxone distribution program*. Vera Institute of Justice. https://www.vera.org/publications/corrections-responses-to-opioid-epidemic-new-york-state

[CR5] Barocas JA, Baker L, Hull SJ, Stokes S, Westergaard RP (2015). High uptake of naloxone-based overdose prevention training among previously incarcerated syringe-exchange program participants. Drug and Alcohol Dependence.

[CR7] Binswanger IA, Stern MF, Deyo RA, Heagerty PJ, Cheadle A, Elmore JG, Koepsell TD (2007). Release from prison—A high risk of death for former inmates. New England Journal of Medicine.

[CR6] Binswanger IA, Blatchford PJ, Mueller SR, Stern MF (2013). Mortality after prison release: Opioid overdose and other causes of death, risk factors, and time trends from 1999 to 2009. Annals of Internal Medicine.

[CR9] Centers for Disease Control and Prevention (2022, May 3). *Guidance on prevention and management of Coronavirus disease 2019 (COVID-19) in correctional and detention facilities*. https://www.cdc.gov/coronavirus/2019-ncov/community/correction-detention/guidance-correctional-detention.html

[CR8] Centers for Disease Control and Prevention (2020, December 17). *Increase in fatal drug overdoses across the United States driven by synthetic opioids before and during the COVID-19 pandemic*. Health Alert Network (HAN). https://emergency.cdc.gov/han/2020/han00438.asp

[CR10] Chandler RK, Villani J, Clarke T, McCance-Katz EF, Volkow ND (2020). Addressing opioid overdose deaths: The vision for the HEALing communities Study. Drug and Alcohol Dependence.

[CR11] Collins AB, Ndoye CD, Arene-Morley D, Marshall BDL (2020). Addressing co-occurring public health emergencies: The importance of naloxone distribution in the era of COVID-19. International Journal of Drug Policy.

[CR12] Curtis M, Dietze P, Aitken C, Kirwan A, Kinner SA, Butler T, Stoové M (2018). Acceptability of prison-based take-home naloxone programmes among a cohort of incarcerated men with a history of regular injecting drug use. Harm Reduction Journal.

[CR13] Davidson PJ, Wagner KD, Tokar PL, Scholar S (2019). Documenting need for naloxone distribution in the Los Angeles County jail system. Addictive Behaviors.

[CR14] Degenhardt L, Grebely J, Stone J, Hickman M, Vickerman P, Marshall BDL, Bruneau J, Altice FL, Henderson G, Rahimi-Movaghar A, Larney S (2019). Global patterns of opioid use and dependence: Harms to populations, interventions, and future action. The Lancet.

[CR15] Donelan CJ, Hayes E, Potee RA, Schwartz L, Evans EA (2021). COVID-19 and treating incarcerated populations for opioid use disorder. Journal of Substance Abuse Treatment.

[CR16] Drabinski E, Rabina D (2015). Reference services to incarcerated people, part I: Themes emerging from answering reference questions from prisons and jails. Reference and User Services Quarterly.

[CR17] El-Bassel N, Jackson RD, Samet J, Walsh SL (2020). Introduction to the special issue on the HEALing communities Study. Drug and Alcohol Dependence.

[CR18] Fagan AA, Bumbarger BK, Barth RP, Bradshaw CP, Cooper BR, Supplee LH, Walker DK (2019). Scaling up evidence-based interventions in US public systems to prevent behavioral health problems: Challenges and opportunities. Prevention Science.

[CR19] Farrell M, Marsden J (2008). Acute risk of drug-related death among newly released prisoners in England and Wales. Addiction.

[CR20] Faust JS, Du C, Mayes KD, Li SX, Lin Z, Barnett ML, Krumholz HM (2021). Mortality from drug overdoses, homicides, unintentional injuries, motor vehicle crashes, and suicides during the pandemic, March-August 2020. Jama.

[CR21] Fazel S, Yoon IA, Hayes AJ (2017). Substance use disorders in prisoners: An updated systematic review and meta-regression analysis in recently incarcerated men and women. Addiction.

[CR22] Feldstein AC, Glasgow RE (2008). A practical, robust implementation and sustainability model (PRISM) for integrating research findings into practice. The Joint Commission Journal on Quality and Patient Safety.

[CR23] Friedman J, Akre S (2021). COVID-19 and the drug overdose crisis: Uncovering the deadliest months in the United States, January–July 2020. American Journal of Public Health.

[CR24] Giglio RE, Li G, DiMaggio CJ (2015). Effectiveness of bystander naloxone administration and overdose education programs: A meta-analysis. Injury Epidemiology.

[CR25] Grella CE, Ostlie E, Scott CK, Dennis ML, Carnevale J, Watson DP (2021). A scoping review of factors that influence opioid overdose prevention for justice-involved populations. Substance Abuse Treatment Prevention and Policy.

[CR26] Harris PA, Taylor R, Thielke R, Payne J, Gonzalez N, Conde JG (2009). Research electronic data capture (REDCap)—A metadata-driven methodology and workflow process for providing translational research informatics support. Journal of Biomedical Informatics.

[CR27] Hedegaard, H., Miniño, A., Spencer, M., & Warner, M. (2021). *Drug overdose deaths in the United States, 1999–2020* (Data Brief No. 428). National Center for Health Statistics. https://stacks.cdc.gov/view/cdc/112340 34978529

[CR28] Horton M, McDonald R, Green TC, Nielsen S, Strang J, Degenhardt L, Larney S (2017). A mapping review of take-home naloxone for people released from correctional settings. International Journal of Drug Policy.

[CR29] Hsieh HF, Shannon SE (2005). Three approaches to qualitative content analysis. Qualitative Health Research.

[CR30] Huhn AS, Strain EC, Jardot J, Turner G, Bergeria CL, Nayak S, Dunn KE (2022). Treatment disruption and childcare responsibility as risk factors for drug and alcohol use in persons in treatment for substance use disorders during the COVID-19 crisis. Journal of Addiction Medicine.

[CR31] Institution for Health Metrics and Evaluation (IHME) (2021). *Global Health Data Exchange*. Institution for Health Metrics and Evaluation (IHME). http://ghdx.healthdata.org/gbd-results-tool?params=gbd-api-2019-permalink/c0b6f59535bbc77251e1aac20c7241ab

[CR32] Kim H, Hughes E, Cavanagh A, Norris E, Gao A, Bondy SJ, McLeod KE, Kanagalingam T, Kouyoumdjian FG (2022). The health impacts of the COVID-19 pandemic on adults who experience imprisonment globally: A mixed methods systematic review. PLOS ONE.

[CR33] Knudsen HK, Drainoni ML, Gilbert L, Huerta TR, Oser CB, Aldrich AM, Campbell ANC, Crable EL, Garner BR, Glasgow LM, Goddard-Eckrich D, Marks KR, McAlearney AS, Oga EA, Scalise AL, Walker DM (2020). Model and approach for assessing implementation context and fidelity in the HEALing communities Study. Drug and Alcohol Dependence.

[CR34] Knudsen HK, Freeman P, Oyler D, Oser CB, Walsh SL (2023). Scaling up overdose education and naloxone distribution in Kentucky: A hub with many spokes model. Addiction Science & Clinical Practice.

[CR35] Krausz RM, Westenberg JN, Ziafat K (2021). The opioid overdose crisis as a global health challenge. Current Opinion in Psychiatry.

[CR36] Krawczyk N, Picher CE, Feder KA, Saloner B (2017). Only one in twenty justice-referred adults in specialty treatment for opioid use receive methadone or buprenorphine. Health Affairs.

[CR37] Krinsky CS, Lathrop SL, Brown P, Nolte KB (2009). Drugs, detention, and death: A study of the mortality of recently released prisoners. The American Journal of Forensic Medicine and Pathology.

[CR38] Leip LA, Stinchcomb JB (2013). Should I stay or should I go? Job satisfaction and turnover intent of jail staff throughout the United States. Criminal Justice Review.

[CR39] LeMasters K, Brinkley-Rubinstein L, Maner M, Peterson M, Nowotny K, Bailey Z (2022). Carceral epidemiology: Mass incarceration and structural racism during the COVID-19 pandemic. The Lancet Public Health.

[CR40] Lim S, Seligson AL, Parvez FM, Luther CW, Mavinkurve MP, Binswanger IA, Kerker BD (2012). Risks of drug-related death, suicide, and homicide during the immediate post-release period among people released from New York City jails, 2001–2005. American Journal of Epidemiology.

[CR41] Mark TL, Gibbons B, Barnosky A, Padwa H, Joshi V (2021). Changes in admissions to specialty addiction treatment facilities in California during the COVID-19 pandemic. JAMA Network Open.

[CR42] Maruschak, L. M., Berzofsky, M., & Unangst, J. (2015). *Medical problems of state and federal prisoners and jail inmates, 2011-12* (Special Report NCJ 248491). US Department of Justice, Office of Justice Programs, Bureau of Justice. https://www.bjs.gov/content/pub/pdf/mpsfpji1112.pdf

[CR43] McDonald R, Campbell ND, Strang J (2017). Twenty years of take-home naloxone for the prevention of overdose deaths from heroin and other opioids—conception and maturation. Drug and Alcohol Dependence.

[CR44] Merrall EL, Kariminia A, Binswanger IA, Hobbs MS, Farrell M, Marsden J, Hutchinson SJ, Bird SM (2010). Meta-analysis of drug-related deaths soon after release from prison. Addiction.

[CR45] Novisky, M. A., Nowotny, K. M., Jackson, D. B., Testa, A., & Vaughn, M. G. (2021). Incarceration as a fundamental social cause of health inequalities: Jails, prisons and vulnerability to COVID-19. *The British Journal of Criminology*, azab023. 10.1093/bjc/azab023

[CR46] Nowotny K, Bailey Z, Omori M, Brinkley-Rubinstein L (2020). COVID-19 exposes need for progressive criminal justice reform. American Journal of Public Health.

[CR47] Prescription Drug Abuse Policy System (PDAPS) (2022, January 1). *Naloxone overdose prevention laws*. https://pdaps.org/datasets/laws-regulating-administration-of-naloxone-1501695139

[CR48] Ranapurwala SI, Shanahan ME, Alexandridis AA, Proescholdbell SK, Naumann RB, Edwards Jr D, Marshall SW (2018). Opioid overdose mortality among former North Carolina inmates: 2000–2015. American Journal of Public Health.

[CR49] Rogers, E. M. (2003). *Diffusion of innovations* (5th ed.). Free.

[CR50] Sander G, Shirley-Beavan S, Stone K (2019). The global state of harm reduction in prisons. Journal of Correctional Health Care.

[CR51] Slavova S, Quesinberry D, Hargrove S, Rock P, Brancato C, Freeman PR, Walsh SL (2021). Trends in drug overdose mortality rates in Kentucky, 2019–2020. JAMA Network Open.

[CR53] Smart R, Pardo B, Davis CS (2021). Systematic review of the emerging literature on the effectiveness of naloxone access laws in the United States. Addiction (Abingdon England).

[CR52] Sprague Martinez L, Rapkin BD, Young A, Freisthler B, Glasgow L, Hunt T, Salsberry PJ, Oga EA, Bennet-Fallin A, Plouck TJ, Drainoni ML, Freeman PR, Surratt H, Gulley J, Hamilton GA, Bowman P, Roeber CA, El-Bassel N, Battaglia T (2020). Community engagement to implement evidence-based practices in the HEALing communities study. Drug and Alcohol Dependence.

[CR54] Stone, K., & Shirley-Beavan, S. (2018). *The global state of harm reduction 2018* [Report]. Harm Reduction International. https://apo.org.au/node/210261 10.1177/1078345819837909 31084277

[CR55] Tanz, L. J., Dinwiddie, A. T., Snodgrass, S., O’Donnell, J., Mattson, C. L., & Davis, N. L. (2022). *Drug overdose deaths in 28 states and the District of Columbia: 2020 data from the State Unintentional Drug Overdose Reporting System*. Centers for Disease Control and Prevention. SUDORS Data Brief No. 2https://stacks.cdc.gov/view/cdc/120987

[CR56] Townsend T, Blostein F, Doan T, Madson-Olson S, Galecki P, Hutton DW (2020). Cost-effectiveness analysis of alternative naloxone distribution strategies: First responder and lay distribution in the United States. International Journal of Drug Policy.

[CR57] Turney K, Conner E (2019). Jail incarceration: A common and consequential form of criminal justice contact. Annual Review of Criminology.

[CR70] U.S. Food & Drug Administration. (2023, March 30). *FDA approves first over-the-counter naloxone nasal spray*. [https://www.fda.gov/news-events/press-announcements/fda-approves-first-over-counter-naloxone-nasal-spray].

[CR59] United States Department of Agriculture (USDA) Economic Research Service (ERS) (2020, December 10). *Rural-urban continuum codes*. https://www.ers.usda.gov/data-products/rural-urban-continuum-codes.aspx

[CR58] United Nations News (2021, March 9). Impact of COVID-19 ‘heavily felt’ by prisoners globally: UN expert | UN News. *United Nations News*. https://news.un.org/en/story/2021/03/1086802

[CR60] Vera (2022, March 24). *Incarceration trends: Kentucky*. https://trends.vera.org/state/KY

[CR61] Wallace, M., Hagan, L., Curran, K., Williams, S., Handanagic, S., & Marlow, M. (2020). COVID-19 in correctional and detention facilities—United States, February–April 2020. *MMWR Morbidity and Mortality Weekly Report*, *69*. 10.15585/mmwr.mm6919e1 10.15585/mmwr.mm6919e1 32407300

[CR62] Walsh SL, El-Bassel N, Jackson RD, Samet JH, Aggarwal M, Aldridge AP, Baker T, Barbosa C, Barocas JA, Battaglia TA, Beers D, Bernson D, Bowers-Sword R, Bridden C, Brown JL, Bush HM, Bush JL, Button A, Campbell ANC, Chandler RK (2020). The HEALing (helping to End Addiction Long-Term SM) communities Study: Protocol for a cluster randomized trial at the community level to reduce opioid overdose deaths through implementation of an integrated set of evidence-based practices. Drug and Alcohol Dependence.

[CR63] Waly, G. F., Ghebreyesus, T. A., Byanyima, W., & Bachelet, M. (2020, May 13). *UNODC, WHO, UNAIDS and OHCHR joint statement on COVID-19 in prisons and other closed settings*. World Health Organization. https://www.who.int/news/item/13-05-2020-unodc-who-unaids-and-ohchr-joint-statement-on-covid-19-in-prisons-and-other-closed-settings

[CR64] Weiner BJ, Amick H, Lee SYD (2008). Conceptualization and measurement of organizational readiness for change: A review of the literature in health services research and other fields. Medical Care Research and Review.

[CR65] Wenger LD, Showalter D, Lambdin B, Leiva D, Wheeler E, Davidson PJ, Coffin PO, Binswanger IA, Kral AH (2019). Overdose education and naloxone distribution in the San Francisco County jail. Journal of Correctional Health Care.

[CR66] Williams NJ, Wolk CB, Becker-Haimes EM, Beidas RS (2020). Testing a theory of strategic implementation leadership, implementation climate, and clinicians’ use of evidence-based practice: A 5-year panel analysis. Implementation Science.

[CR67] Winhusen T, Walley A, Fanucchi LC, Hunt T, Lyons M, Lofwall M, Brown JL, Freeman PR, Nunes E, Beers D, Saitz R, Stambaugh L, Oga EA, Herron N, Baker T, Cook CD, Roberts MF, Alford DP, Starrels JL, Chandler RK (2020). The opioid-overdose reduction continuum of Care Approach (ORCCA): Evidence-based practices in the HEALing communities Study. Drug and Alcohol Dependence.

[CR68] Winter RJ, Young JT, Stoové M, Agius PA, Hellard ME, Kinner SA (2016). Resumption of injecting drug use following release from prison in Australia. Drug and Alcohol Dependence.

[CR69] Young AM, Brown JL, Hunt T, Martinez LSS, Chandler R, Oga E, Winhusen TJ, Baker T, Battaglia T, Bowers-Sword R, Button A, Fallin-Bennett A, Fanucchi L, Freeman P, Glasgow LM, Gulley J, Kendell C, Lofwall M, Lyons MS, Walsh SL (2022). Protocol for community-driven selection of strategies to implement evidence-based practices to reduce opioid overdoses in the HEALing communities Study: A trial to evaluate a community-engaged intervention in Kentucky, Massachusetts, New York and Ohio. British Medical Journal Open.

